# Toward Mechanically Robust Crosslinked Elastomers through Phase Transfer Agent Tuning the Solubility of Zn^2+^ in the Organic Phase

**DOI:** 10.3390/polym14061234

**Published:** 2022-03-18

**Authors:** Shuang Liu, Xin-Yao Quan, Hao-Ran Wang, Shuangquan Liao, Ming-Chao Luo

**Affiliations:** 1 Key Laboratory of Advanced Materials of Tropical Island Resources of Ministry of Education, Natural Rubber Cooperative Innovation Center of Hainan Province & Ministry of Education of PRC, School of Materials Science and Engineering, Hainan University, Haikou 570228, China; 13515328463@163.com (S.L.); 20190481310115@hainanu.edu.cn (X.-Y.Q.); wanghaoran199994@163.com (H.-R.W.); 2 Key Laboratory of Carbon Fiber and Functional Polymers, Beijing University of Chemical Technology, Ministry of Education, Beijing 100000, China

**Keywords:** elastomers, phase transfer agent, crosslinking process, ZnO content, mechanical properties

## Abstract

Zinc oxide (ZnO), which is toxic to aquatic organisms, is widely used as an activator in the rubber industry. The reduction of ZnO content is one of the efficient ways to tackle ecological environment impacts induced by ZnO. However, the incompatibility between Zn^2+^ and organic matrix inhibits the solubility and activity of Zn^2+^ in the organic matrix, causing the heavy use of ZnO. This work develops a phase transfer agent with Zn^2+^-philic structure and oleophilic structure to increase the solubility of Zn^2+^ in the organic matrix. The phase transfer agent and Zn^2+^ form coordination interactions, while the hydrophobic chains of phase transfer agent and organic matrix form hydrophobic interactions. The above two interactions improve the solubility and activity of Zn^2+^ in the organic matrix, contributing to the formation of crosslinking network. Through the phase transfer agent strategy, we obtain the mechanically robust elastomers, and the samples with low ZnO content still maintain the superior properties. This work provides an efficient way to reduce ZnO content without sacrificing the performance of elastomers.

## 1. Introduction

Elastomers, such as natural rubber (NR) and polyisoprene, are widely used in the field of engineering materials and functional materials [1,2,3,4,5]. Almost all rubber products go through the crosslinking (vulcanization) process, because such a process endows materials with three-dimensional network and superior properties. In the present crosslinking (vulcanization) system, zinc oxide (ZnO), as an important activator, contributes to the formation of crosslinking network [6,7,8,9,10]. However, the European Commission has classified ZnO as toxic to aquatic organisms and controlled its application in rubber technology [11]. The reduction of ZnO content is one of the efficient ways to tackle ecological environment and human health impacts induced by ZnO. According to the previous work [12], ZnO content is closely related to ZnO activity. Organic polymer chains and metal ion (Zn^2+^) are often incompatible. Such incompatibility inhibits the activity of Zn^2+^ in the organic matrix and suppresses the formation of crosslinking network, causing the heavy use of ZnO in the matrix. To decrease the content of ZnO, we still face a tricky problem—how to increase the solubility and activity of ZnO in the organic matrix.

In the conventional crosslinking (vulcanization) process, ZnO can react with stearic acid to form the active zinc chelates. It is a dinuclear bridging bidentate zinc/stearate complex [13]. The zinc/stearate complex first reacts to form a chelate. Subsequently, such chelates react with sulfur to form polysulfide intermediates containing Zn^2+^, which further weaken S-S bonds and catalyze the crosslinking reaction [14,15,16]. However, due to the incompatibility between Zn^2+^ and organic matrix, it is difficult for Zn^2+^ to enter the rubber matrix, and the catalytic activity of Zn^2+^ is limited in the rubber matrix. Moreover, the previous works have proved that Zn^2+^ exists in the form of a dinuclear bridging bidentate zinc/stearate intermediate [17,18]. Such high steric hindrance of bidentate intermediate inhibits the catalytic activity of Zn^2+^ in the organic phase and then affects crosslinking reaction [18,19]. To increase the activity of Zn^2+^ in the matrix, the increase of Zn^2+^ solubility in the matrix is an important factor. Our group designs a phase transfer agent, which is comprised of Zn^2+^-philic structure and oleophilic structure. Such a phase transfer agent is beneficial for the entrance of Zn^2+^ into the organic matrix, which further improves the activity of Zn^2+^ in the matrix.

In this work, we develop a phase transfer agent strategy to increase the solubility and activity of Zn^2+^ in the organic phase and obtain the mechanically robust crosslinked elastomers. Through the design of phase transfer agent, this work obtains the high-performance elastomers with low ZnO content, which provides an efficient and green way to decrease the content of ZnO in the crosslinking process.

## 2. Materials and Methods

### 2.1. Materials

NR latex from clone RRIM600 was provided by China Hainan Rubber Industry Group Co., Ltd. (Hainan, China). Vulcanization additives, such as stearic acid, ZnO, accelerator 2-mercaptobenzothiazole (M), and sulfur, were industrial grade. Sodium dodecyl sulfate (SDS, 99%), propionamide, and 2-piperidone (Pip, 98%) were purchased from Aladdin Bio-Chem Technology Co., Ltd. (Shanghai, China).

### 2.2. Preparation

We diluted the NR latex to a mass fraction of 30% and added 0.5% SDS to it. SDS was used as a surfactant, and its addition increased the stability of the fresh NR latex [20,21,22,23]. The fresh NR latex required the treatment of high-speed centrifugation (12,000 rpm, 60 min) and we obtained the centrifugated NR (CNR). Next, we added water to dilute it to 30% (*w*/*w*). The resultant latex was spread on a glass plate and dried naturally to constant weight under room temperature. CNR was blended with vulcanization additives and phase transfer agent (Pip) on a two-roll mill. The formulation is as follows: sample 100 phr, sulfur 3 phr, ZnO 5 phr, steric acid 0.5 phr, accelerator M 0.7 phr, and Pip 0 phr-3 phr. According to the optimum curing time, the compounds were subjected to compression at 145 °C.

### 2.3. Characterization

The crosslinking (vulcanization) process was investigated by an oscillating disc rheometer (GOTECH M-3000AU) (Qingdao, China). After pre-heating the disc to a temperature of 145 °C, 6 g of sample was inserted between the two discs and the torque was monitored as a function of time.

Differential scanning calorimeter (DSC) was measured at different heating rates (3, 10, 15, and 20 °C/min) on TA Instruments Q100 (New Castle, DE, USA). The weight of each sample was in the range of 5–7 mg and the measurements were under nitrogen atmosphere.

We used model compound to analyze the interactions of system. ZnO, accelerator, and phase transfer agent (for example, propionamide) were mixed together, which was further subjected to compression at 145 °C for 10 min. The mixture (ZnO, accelerator, and phase transfer agent) and phase transfer agent were investigated by PerkinElmer Fourier transform infrared (FTIR) spectrometer (PerkinElmer, Waltham, MA, USA) at room temperature, respectively. We used KBr for transmission spectroscopy testing. The resolution was 4 cm^−1^. The wavenumber range was from 4000 cm^−1^ to 400 cm^−1^ and the number of scans was 32. Moreover, the mixture and phase transfer agent were also researched by X-ray photoelectron spectroscopy (XPS), respectively. XPS measurements were carried out on a Kratos Axis Supra Instrument (Thermo Fisher Scientific, Waltham, MA, USA) with Al Kα source (1486.6 eV) operating at 600 W. The vacuum in operation was approximately 8 × 10^−9^ Torr. High-resolution scans of the O1s, C1s, and N1s XPS peaks were recorded.

Stress–strain curves were obtained using a GOTECH AI-3000 testing machine (Qingdao, China) with an extension rate of 500 mm/min at room temperature. The specimen was a dumbbell-shaped flake with central dimensions of 25 mm × 6 mm × 1 mm. The tear strength was obtained using the same instrument.

Crosslinking density was determined by equilibrium swelling method. The samples were swollen in toluene at room temperature for 7 days and then solvent was removed quickly from the swollen sample surface using filter paper. The samples were immediately weighed and dried to constant weight in a vacuum oven at 80 °C. Crosslinking density was calculated by the following equations:

$$-ln\left(1-\phi_{r}\right)-\phi_{r}-\chi_{r}{\phi_{r}}^{2}=nV_{0}\left(\phi_{r}^{1/3}-\frac{1}{2}\phi_{r}\right)$$

$$M_{c}=\frac{\rho }{n}$$

where *ϕ_r_* is the polymer volume fraction in the swollen network, *V*_0_ is the molar volume of the solvent (106.2 mL/mol for toluene), *χ_r_* is the Flory–Huggins polymer–solvent interaction term (0.393 for NR/toluene), *n* is the average number of movable chain segments per unit volume (mol/mL), *M_c_* is the average mass of network chains, and *ρ* is the density of NR (0.913 g/mL for NR).

Temperature-dependent curves of loss factor (tanδ) were measured in tensile mode on a TA dynamic mechanical analyzer (DMA) 850 (TA Instruments, New Castle, DE, USA) with a gas cooling accessory under nitrogen atmosphere. The experiment was performed at a heating rate of 3 °C/min and a frequency of 1 Hz. The temperature range was from −100 °C to 100 °C. In all cases, a preload force of 0.01 N was applied.

## 3. Results

### 3.1. Changes in Crosslinking Process Induced by Phase Transfer Agent

**Figure 1:**
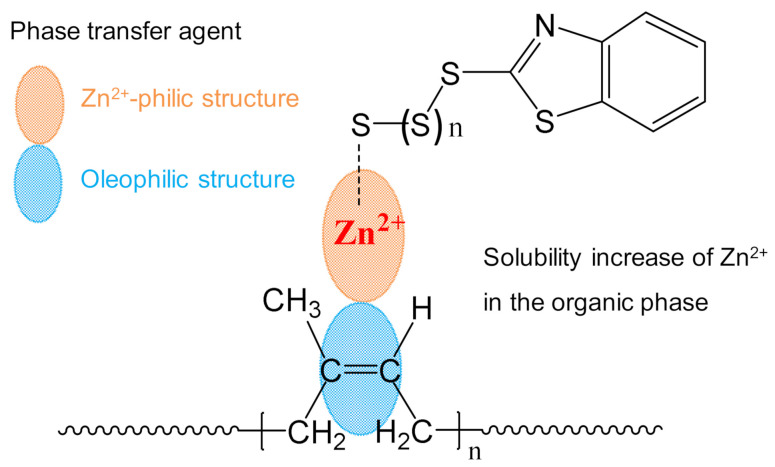
Conceptual scheme of phase transfer agent tuning the solubility of Zn^2+^ in the organic phase.

Hydrophilic Zn^2+^ and oleophilic rubber chains are incompatible in thermodynamics, limiting the catalytic ability of Zn^2+^ in the organic phase. In this work, our group designs a phase transfer agent to increase the solubility of Zn^2+^ in the organic polymer, as shown in Figure 1. Our designed phase transfer agent is comprised of Zn^2+^-philic structure and oleophilic structure. The previous work has proved that amide bonds and Zn^2+^ are prone to form coordination interactions [24]. The Zn^2+^-philic structure contains amide bond, which constructs the coordination interactions between phase transfer agent and Zn^2+^. Another structure is the alkyl chain, which provides hydrophobic interactions between phase transfer agent and organic polymer phase. Based on the above two interactions among phase transfer agent, Zn^2+^, and organic phase, the solubility of Zn^2+^ in the matrix increases.

**Figure 2:**
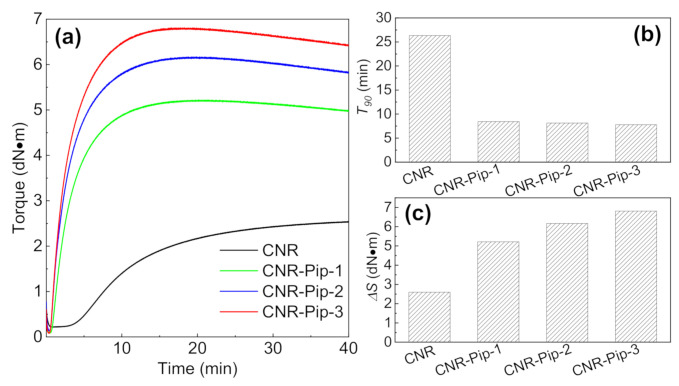
Changes in vulcanization process after the addition of phase transfer agent. (**a**) Vulcanization curves of CNR, CNR-Pip-1, CNR-Pip-2, and CNR-Pip-3. (**b**) *T*_90_ comparison among CNR, CNR-Pip-1, CNR-Pip-2, and CNR-Pip-3. (**c**) *ΔS* comparison among CNR, CNR-Pip-1, CNR-Pip-2, and CNR-Pip-3.

Based on the structure characteristic of phase transfer agent, Piperidone (Pip) is chosen as a phase transfer agent. We denote CNR with Pip as CNR-Pip-*x*, where *x* is the weight fraction of Pip. The introduction of phase transfer agent has an obvious impact on crosslinking process (Figure 2a). For example, with the increase of Pip, CNR-Pip owns the shorter optimum vulcanization time (*T*_90_) and the larger difference between minimum torque and maximum torque (*ΔS*), as shown in Figure 2b,c.

### 3.2. Effect of Phase Transfer Agent on Crosslinking Structure

**Figure 3:**
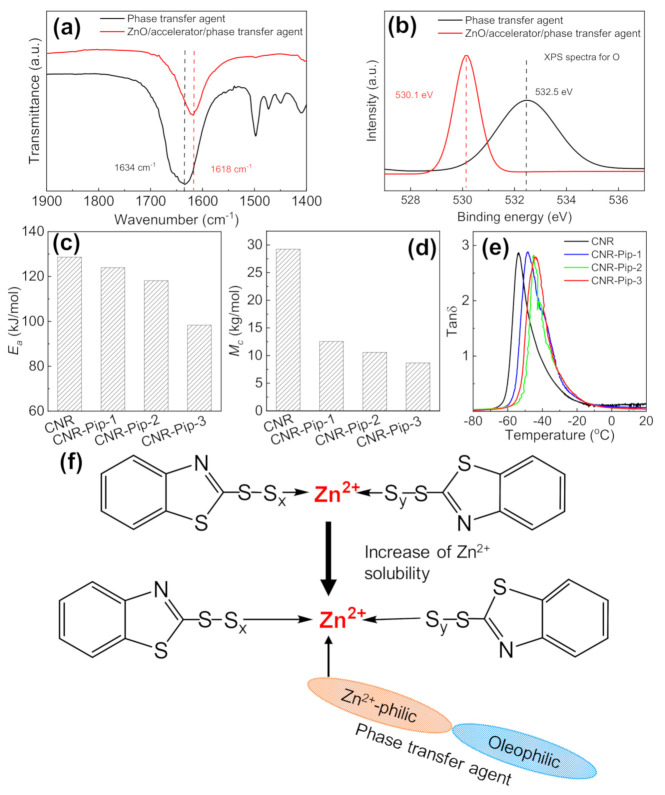
Changes in crosslinking structure after the addition of phase transfer agent. (**a**) FTIR spectra of phase transfer agent and ZnO/accelerator/phase transfer agent. (**b**) XPS spectra of phase transfer agent and ZnO/accelerator/phase transfer agent. (**c**) *E_a_* of CNR, CNR-Pip-1, CNR-Pip-2, and CNR-Pip-3. (**d**) *M_c_* of CNR, CNR-Pip-1, CNR-Pip-2, and CNR-Pip-3. (**e**) Temperature dependence of tanδ curves for CNR, CNR-Pip-1, CNR-Pip-2, and CNR-Pip-3. (**f**) Schematic illustration of Zn^2+^ solubility increase in the matrix by phase transfer agent.

The solubility and activity of Zn^2+^ in the organic phase are the important factors in determining crosslinking process and the formation of crosslinking network. To explore the solubility of Zn^2+^ in the organic matrix, we first investigated the interactions among phase transfer agent, organic matrix, and Zn^2+^. Through the investigation of model compound ZnO/accelerator/phase transfer agent by FTIR and XPS, we verify the interactions between phase transfer agent and Zn^2+^ (Figure 3a,b). The complete FTIR spectra is shown in Figure S1. The characteristic peak of amide bonds is at 1634 cm^−1^, as shown in Figure 3a. After the addition of phase transfer agent, such characteristic peak shifts toward lower wavenumbers. Moreover, the coordination interactions between phase transfer agent and Zn^2+^ are also demonstrated by XPS (Figure 3b). The position shift of elemental binding energy depends on the changes of electron cloud density [25]. After the mixture of ZnO, accelerator, and phase transfer agent, the characteristic peak (532.5 eV) shifts toward 530.1 eV, suggesting the formation of coordination interaction. The results of FTIR and XPS prove the interaction construction between phase transfer agent and Zn^2+^. Besides, the alkyl chain of phase transfer agent provides the hydrophobic interactions between phase transfer agent and organic phase. The above coordination interactions and hydrophobic interactions increase the solubility of Zn^2+^ in the organic matrix.

To further investigate the changes in crosslinking process and crosslinking structure by tuning the solubility of Zn^2+^ in the organic polymer matrix, we use DSC to research crosslinking kinetics. DSC is performed to obtain heat flow curves at different heating rates (Figures S2 and S3). According to the previous works [26,27], we extract exothermic peak data and fit the data points to obtain activation energy (*E_a_*) from the slope:

$$E_{a}=-R\frac{dln\beta }{d\left(1/T\right)}$$

where *β* is the heating rate, *T* is the peak temperature of exothermal peak, and *R* is the gas constant. The increase of Zn^2+^ solubility in the matrix by phase transfer agent improves the activity of Zn^2+^ in crosslinking reaction, thereby decreasing the *E_a_* of crosslinking process (Figure 3c) and changing the crosslinking process (Figure 2). Moreover, the improvement of Zn^2+^ activity in the matrix by phase transfer agent is beneficial for the formation of crosslinking network, causing the increase of crosslinking density and glass transition temperature (Figure 3d,e). Based on the above analysis, phase transfer agent and Zn^2+^ form coordination interactions, while phase transfer agent and organic matrix form hydrophobic interactions. These two interactions increase the solubility of Zn^2+^ in the matrix and then improve the activity of Zn^2+^ in crosslinking process, as illustrated in Figure 3f. The schematic diagram of 2-piperidone participating in the vulcanization crosslinking process is shown in Figure S4.

### 3.3. Effect of Phase Transfer Agent on Mechanical Properties

**Figure 4:**
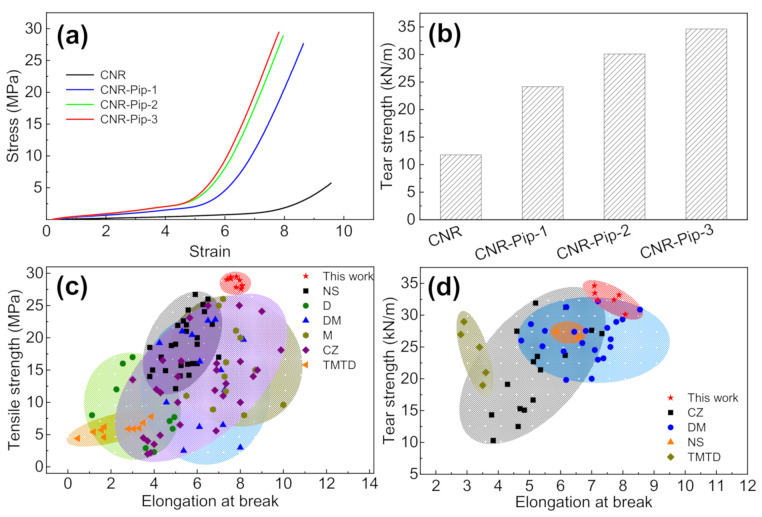
Changes in mechanical properties upon the addition of phase transfer agent. (**a**) Stress–strain curves of CNR, CNR-Pip-1, CNR-Pip-2, and CNR-Pip-3. (**b**) Tear strength of CNR, CNR-Pip-1, CNR-Pip-2, and CNR-Pip-3. (**c**) Comparison of tensile strength and elongation at break among this work and the elastomers with different accelerators. (**d**) Comparison of tear strength and elongation at break among this work and the elastomers with different accelerators.

To further explore the mechanical properties of samples with phase transfer agent, we adopt uniaxial tensile tests on a tensile machine. After the addition of phase transfer agent, CNR-Pip samples exhibit a 5.14-fold increase in tensile strength (from 5.73 MPa to 29.4 MPa) and a 2.94-fold increase in tear strength (from 11.8 kN/m to 34.6 kN/m), as shown in Figure 4a,b. Such dramatic improvements in mechanical properties are ascribed to the promotion of crosslinking network formation by the increase of Zn^2+^ solubility in the organic matrix.

In the present crosslinking process, the performance regulations of elastomers depend on crosslinking (vulcanization) systems (for example, accelerator). The conventional crosslinking (vulcanization) systems show a lack of tuning Zn^2+^ solubility in the organic matrix, which limits the further improvements in mechanical properties. Through the phase transfer agent strategy, we can increase the solubility of Zn^2+^ in the organic phase and then further promote the mechanical properties of crosslinked elastomers. To highlight the importance of phase transfer agent to properties of crosslinked elastomers, we compare tensile strength, tear strength, and elongation at break among this work and the elastomers with different accelerators in Figure 4c,d. The data of elastomers with different accelerators are from the previous works, containing N-cyclohexyl-2-benzothiazolesulfenamide (CZ) [28,29,30,31,32,33,34,35,36,37,38,39,40,41,42,43,44,45,46,47,48], diphenyl guanidine (D) [49,50], dibenzothiazyl disulfide (DM) [51,52,53,54,55,56,57], 2-mercaptobenzothiazole (M) [2,58,59,60,61,62,63,64,65], N-tert-butylbenzothiazole-2-sulphenamide (NS) [30,66,67,68,69,70,71], and tetra methyl thiuram disulfide (TMTD) [30,72,73,74]. In Figure 4c,d, our work exhibits a superiority in mechanical properties. The improvements of Zn^2+^ solubility and activity in the organic matrix contribute to the formation of crosslinking network and further enhance the performances of conventional crosslinked elastomers.

### 3.4. Changes in Properties upon Lowering ZnO Content

**Figure 5:**
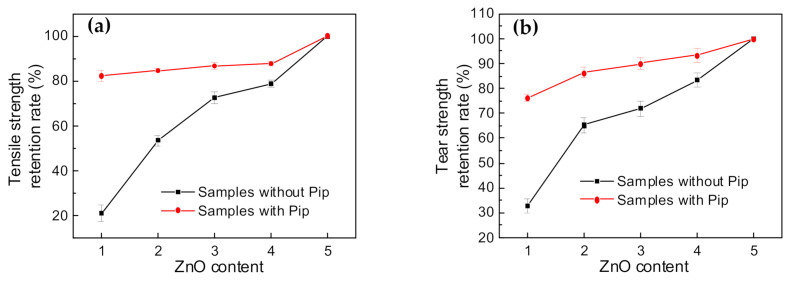
Changes in mechanical properties with low ZnO content. (**a**) Comparison of tensile strength retention rate with different ZnO contents for the samples with and without phase transfer agent. (**b**) Comparison of tear strength retention rate with different ZnO contents for the samples with and without phase transfer agent.

ZnO plays an important role in crosslinking reaction. However, ZnO is toxic to aquatic organisms. Considering the environment pollution induced by ZnO, our group decreases the content of ZnO through phase transfer agent, improving the solubility of Zn^2+^ in the matrix. As the content of ZnO decreases, the samples without Pip exhibit a significant decrease in crosslinking degree (Figure S5a) and mechanical properties (Figures S6a and S7a). Compared with the samples without phase transfer agent, the samples with phase transfer agent show slight changes in the crosslinking process (Figure S5b) and mechanical properties (Figures S6b and S7b). To clearly illustrate the superiority of our work in reducing ZnO content, we compared the tensile strength and tear strength retention rate among the samples with and without phase transfer agent (Figure 5). In the conventional crosslinking (vulcanization) system, the content of ZnO is 5 phr per 100 phr sample. For the calculation of retention rate, we take the properties of the samples with 5 phr ZnO as a benchmark. The ratio of the sample property with different ZnO contents to that with 5 phr ZnO is the retention rate. As the content of ZnO decreases, the tensile strength and tear strength retention rate of samples without Pip show a dramatic decrease. Pip (phase transfer agent) increases the solubility and activity of Zn^2+^ in the organic matrix, contributing to the formation of crosslinking network. After the addition of Pip, the samples with low ZnO content still maintain superior properties.

## 4. Conclusions

In this work, we develop a phase transfer agent strategy to increase the solubility and activity of Zn^2+^ in the organic matrix. Phase transfer agent and Zn^2+^ form coordination interactions, while phase transfer agent and organic matrix form hydrophobic interactions. Such two interactions increase the solubility of Zn^2+^ in the matrix and then improve the activity of Zn^2+^ in crosslinking process, further contributing to the formation of crosslinking network. Our designed elastomers exhibit the superiority in mechanical properties, compared with the conventionally crosslinked elastomers. Moreover, after the addition of phase transfer agent, the samples with low ZnO content still maintain superior properties. Through phase transfer agent strategy, this work not only obtains the mechanically robust elastomers, but also dramatically reduces the content of ZnO in crosslinking process.

## Data Availability

The data presented in this study are contained within the article.

## Supplementary Materials

The following supporting information can be downloaded at: https://www.mdpi.com/article/10.3390/polym14061234/s1, Figure S1: The complet FTIR spectra of phase transfer agent and ZnO/accelerator/phase transfer agent, Figure S2: Heating flow curves of CNR at different heating rates, Figure S3: Heating flow curves of CNR-Pip samples at different heating rates. (a) CNR-Pip-1. (b) CNR-Pip-2. (c) CNR-Pip-3, Figure S4: The schematic diagram of 2-piperidone participating in the vulcanization crosslinking process of natural rubber, Figure S5: (a) Effect of ZnO content on vulcanization curves for the samples without phase transfer agent (Pip). (b) Effect of ZnO content on vulcanization curves for the samples with phase transfer agent (Pip), Figure S6: (a) Effect of ZnO content on stress–strain curves for samples without phase transfer agent (Pip). (b) Effect of ZnO content on stress–strain curves for samples with phase transfer agent (Pip), Figure S7: (a) Effect of ZnO content on tear strength for samples without phase transfer agent (Pip). (b) Effect of ZnO content on tear strength for samples with phase transfer agent (Pip).
