# Supplementary material for: Toward Mechanically Robust Crosslinked Elastomers through Phase Transfer Agent Tuning the Solubility of Zn2+ in the Organic Phase
Source: Polymers (Basel). 2022 Mar 18;14(6):1234. doi: 10.3390/polym14061234 (PMC8949273; doi:10.3390/polym14061234)
Supplement: Supplementary file 1 [file polymers-14-01234-s001.zip › polymers-1631047-supplementary.pdf]

polymers

MDPI

Article

Effects of Underwater Friction Stir Welding Heat Generation on Residual Stress of AA6068-T6 Aluminum Alloy

Hassanein I. Khalaf [1], Raheem Al-Sabur [1], Mahmoud E. Abdullah [2], Andrzej Kubit [3] and Hamed Aghajani Derazkola [4,*]

[1] Mechanical Department, Engineering College, University of Basrah, Basrah 6100, Iraq; hassanein.khalaf@uobasrah.edu.iq (H.I.K.); raheem.musawel@uobasrah.edu.iq (R.A.-S.)
[2] Mechanical Department, Faculty of Technology and Education, Beni-Suef University, Beni-Suef 62511, Egypt; iec.mahmoud@gmail.com
[3] Department of Manufacturing and Production Engineering, Faculty of Mechanical Engineering and Aeronautics, Rzeszow University of Technology, Al. Powst. Warszawy 8, 35-959 Rzeszów, Poland; akubit@prz.edu.pl
[4] Department of Mechanics, Design and Industrial Management, University of Deusto, Avda Universidades 24, 48007 Bilbao, Spain
* Correspondence: h.aghajani@deusto.es

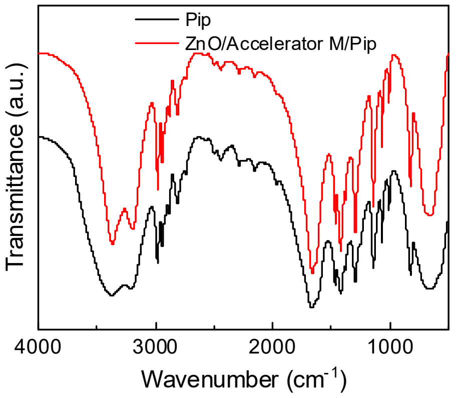

Figure S1. The complet FTIR spectra of phase transfer agent and ZnO/accelerator/phase transfer agent.

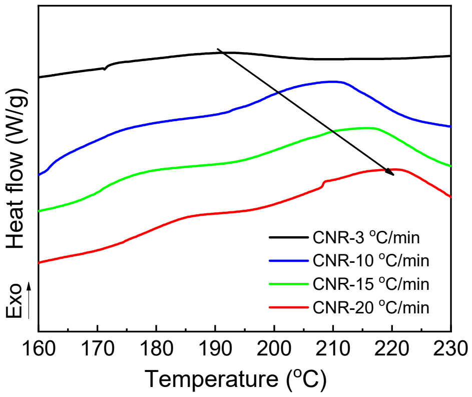

Figure S2. Heating flow curves of CNR at different heating rates.

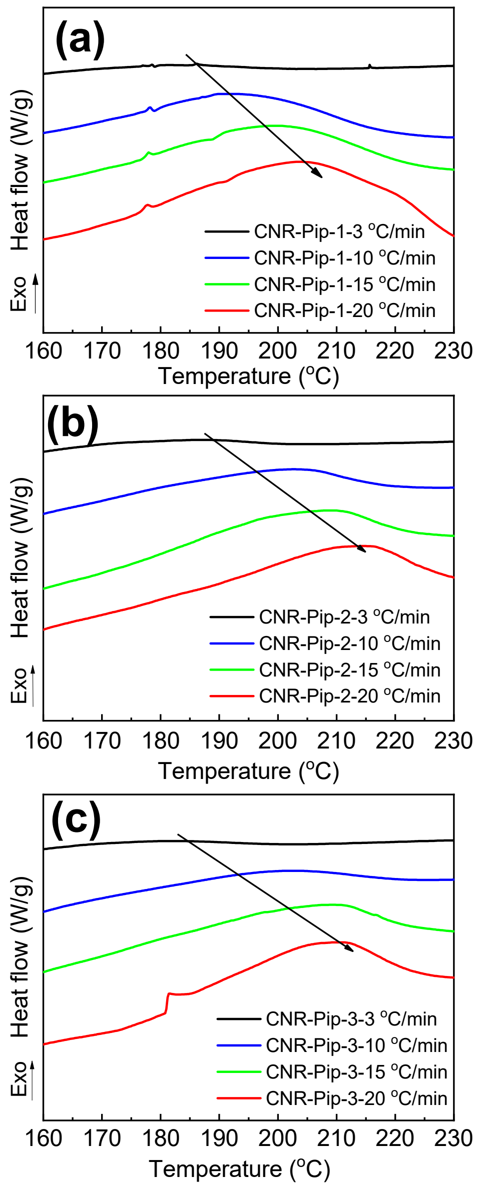

Figure S3. Heating flow curves of CNR-Pip samples at different heating rates. (a) CNR-Pip-1. (b) CNR-Pip-2. (c) CNR-Pip-3.

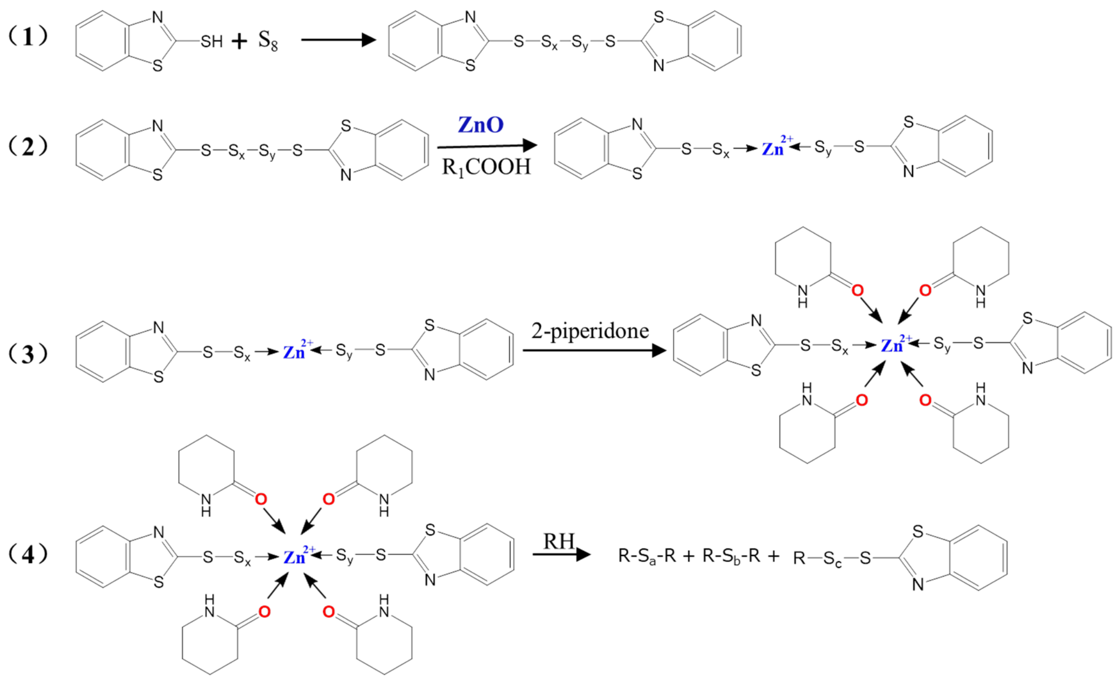

Figure S4. The schematic diagram of 2-piperidone participating in the vulcanization crosslinking process of natural rubber.

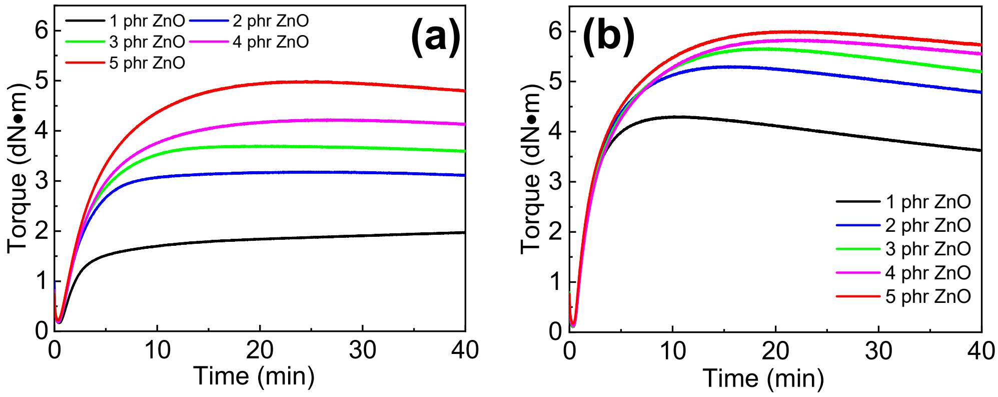

Figure S5. (a) Effect of ZnO content on vulcanization curves for the samples without phase transfer agent (Pip). (b) Effect of ZnO content on vulcanization curves for the samples with phase transfer agent (Pip).

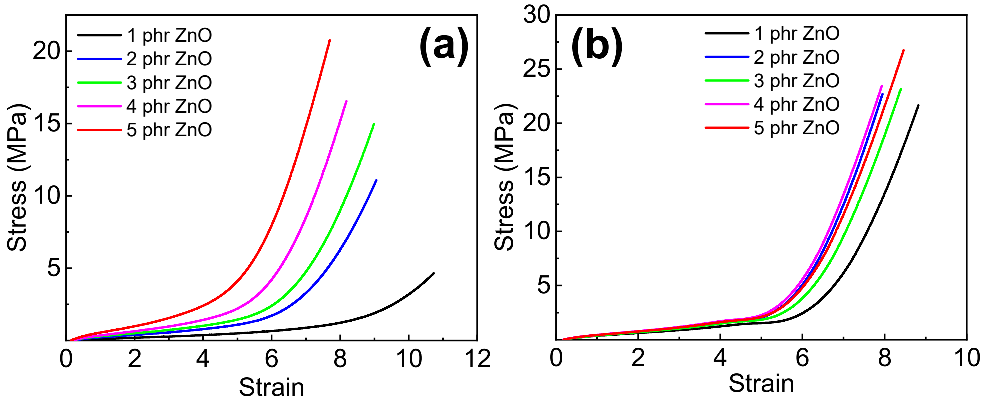

Figure S6. (a) Effect of ZnO content on stress-strain curves for samples without phase transfer agent (Pip). (b) Effect of ZnO content on stress-strain curves for samples with phase transfer agent (Pip).

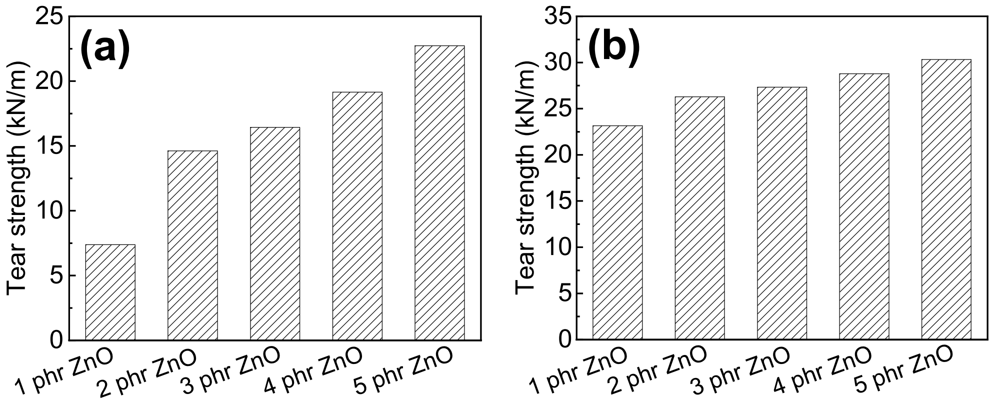

Figure S7. (a) Effect of ZnO content on tear strength for samples without phase transfer agent (Pip). (b) Effect of ZnO content on tear strength for samples with phase transfer agent (Pip).
